# Shifting Determinants of Mortality Risk After Orthotopic Heart Transplantation Identified by Machine Learning

**DOI:** 10.3390/jcdd12120486

**Published:** 2025-12-10

**Authors:** Kinga Bianka Koritsánszky, Rita Szentgróti, Ádám Szijártó, Márton Tokodi, Alexandra Vereb, Andrea Kőszegi, Balázs Sax, Attila Kovács, Béla Merkely, Andrea Székely

**Affiliations:** 1 Heart and Vascular Center, Semmelweis University, H-1122 Budapest, Hungary; kinga.koritsanszky@gmail.com (K.B.K.); szentgrotirita@gmail.com (R.S.); sz.adam1996@gmail.com (Á.S.);; 2 Doctoral School, Semmelweis University, H-1122 Budapest, Hungary; 3 Department of Anesthesiology and Intensive Therapy, Semmelweis University, H-1122 Budapest, Hungary; 4 Department of Oxiology and Emergency Care, Faculty of Health Sciences, Semmelweis University, H-1122 Budapest, Hungary

**Keywords:** heart transplantation, artificial intelligence, risk stratification, explainability

## Abstract

Background: Orthotopic heart transplantation (OHT) remains the gold standard for end-stage heart failure, yet individualized risk assessment for postoperative mortality remains challenging. We aimed to develop and interpret random forest-based models for predicting 30-day and 1-year mortality and to examine whether the key predictors differ between the 30-day and 1-year models. Methods: We analyzed 581 patients who underwent OHT between 2012 and 2024. The 30-day and 1-year mortality rates were 9.9% and 17.6%, respectively. Eighty-seven preoperative and forty-eight postoperative variables were considered as input features for model development. Random forest models were trained and validated using five-fold cross-validation, and explainability was assessed using SHapley Additive exPlanations (SHAP). Results: Using preoperative features only, the random forest models achieved AUCs of 0.62 (95% CI, 0.48–0.75) for 30-day and 0.67 (95% CI, 0.56–0.78) for 1-year mortality. SHAP analysis revealed that early mortality predictions were primarily driven by features reflecting acute physiological stress—hepatic dysfunction, inflammation, and hemodynamic instability—whereas long-term predictions were increasingly influenced by renal function, metabolic reserve, and frailty. Incorporating postoperative features improved performance (AUC 0.98 [95% CI, 0.97–0.99] and 0.86 [95% CI, 0.80–0.92], respectively), with model predictions dominated by the severity and persistence of organ dysfunction: short-term risk driven by hepatic injury, hemodynamic compromise, and critical illness, and long-term risk by sustained hepatic and renal impairment, metabolic resilience, and duration of circulatory support. Conclusions: Random forest models integrating preoperative and immediate postoperative data could predict short- and mid-term mortality after OHT. SHAP analysis demonstrated temporal shifts in the most important predictors, supporting the role of dynamic, data-driven risk assessment in transplant care.

## 1. Introduction

Orthotopic heart transplantation (OHT) remains the standard of care for patients with end-stage heart failure who no longer benefit from pharmacological or device-based therapy [1]. Advances in surgical techniques, immunosuppression, and perioperative management have markedly improved post-transplant survival over recent decades [1]. Nevertheless, morbidity and mortality remain substantial, and outcomes vary widely among recipients. Refining patient selection and improving risk assessment, therefore, remain central goals for optimizing both short- and long-term outcomes [2].

Accurate prognostic evaluation is essential throughout the transplant pathway. It guides eligibility decisions, informs preoperative management, and supports individualized post-transplant care. Inadequate risk stratification may lead to preventable complications, graft failure, or premature mortality, whereas reliable prediction tools enable more equitable donor organ allocation and tailored patient management [3]. As the number of potential candidates continues to grow, the need for robust and clinically applicable predictive models has become increasingly evident.

Conventional risk scores based on multivariable regression have provided important insights but are constrained by linear assumptions, limited input features, and potential overfitting. Such models often fail to capture the complex, nonlinear interactions that characterize clinical reality, resulting in only modest predictive performance [4].

Machine learning (ML) provides an alternative approach that can model multidimensional relationships among diverse clinical, hemodynamic, and biochemical features without requiring prespecified assumptions. ML models have shown promising results in cardiovascular medicine, improving diagnostic accuracy and outcome prediction across a range of applications [4,5,6]. In the context of OHT, these methods may enable more accurate identification of high-risk patients and provide a dynamic view of how the importance of prognostic determinants evolves over time [6].

In this study, we applied ML-based modeling to a large single-center cohort of OHT recipients to predict 30-day and 1-year mortality. Using random forest models with SHapley Additive exPlanations (SHAP), we sought to identify the most important predictors of adverse outcomes and to characterize how their importance evolves between early and late postoperative phases.

## 2. Methods

### 2.1. Database

A retrospective analysis was conducted using the institutional database of adolescent and adult patients who underwent OHT between 30 January 2012 and 23 December 2024 at the Heart and Vascular Center of Semmelweis University (*n* = 581). The study was performed in accordance with the principles outlined in the Declaration of Helsinki, and its protocol was approved by the Regional Ethics Committee (SE RKEB 229/2025).

The database included 87 preoperative features, including baseline demographics, medical history, laboratory results, right heart catheterization data, donor characteristics, immediate preoperative factors, and medications. Further 48 postoperative features were available, including intensive care unit (ICU) and ventilation times, laboratory peak or nadir values depending on the direction of relevant pathological findings, duration and maximum dosage of circulatory support medications, the number of transfusion units administered, and postoperative complications.

### 2.2. Outcomes of Interest

ML models were developed to predict 30-day and 1-year all-cause mortality using either preoperative features alone or all available features (preoperative and postoperative). Data on all-cause mortality [status (dead or alive), date of death] were obtained for all patients by querying Hungary’s National Health Insurance Database on 3 September 2025.

### 2.3. Data Preprocessing and Model Development

We conducted our experiments following our previously published standardized ML framework for data preprocessing, model development, and evaluation [7]. Features with more than 40% missing values were excluded (13 of 87 preoperative variables and 6 of 48 postoperative variables). For the remaining features, missing values in continuous variables were imputed using the mean, whereas categorical variables were imputed using the mode. After imputation, continuous features were standardized using z-score normalization. All preprocessing steps (imputation and standardization) were performed within the inner loop of the nested cross-validation, fitted only on the training split to prevent leakage. Feature selection was performed to identify a subset of 15–25 relevant predictors. To mitigate class imbalance, the Synthetic Minority Oversampling Technique (SMOTE) was applied exclusively to the training portion within each fold of the nested cross-validation pipeline, ensuring that validation and test data remained untouched and avoiding any risk of data leakage [8].

Among commonly used supervised learning approaches, random forest was selected for model development due to its robustness in capturing nonlinear relationships and complex feature interactions and its favorable bias-variance characteristics [9]. This choice was further supported by prior evidence demonstrating its consistent performance and interpretability in clinical prediction tasks involving heterogeneous datasets [4]. A nested cross-validation strategy was employed, consisting of a 5-fold inner cross-validation loop for hyperparameter optimization and a 5-fold outer loop for feature selection, model selection, and performance evaluation. The final model ensemble comprised five classifiers, each trained on a distinct outer training fold and validated on the corresponding hold-out fold. Model performance was primarily assessed using the area under the receiver operating characteristic curve (AUC). Model explainability was examined using SHapley Additive exPlanations (SHAP) values, which quantified both the magnitude and direction of each feature’s contribution to individual predictions [10]. We computed SHAP values for both the 30-day and 1-year models and min–max normalized the absolute SHAP scores to allow direct comparison across predictors. For each approach (i.e., only preoperative or all pre- and postoperative features used), features were ranked by their normalized SHAP values, and a joint feature set was constructed by including (i) the top predictor from the 30-day model, (ii) the top 12 predictors from the 30-day model (forming the core short-term signature), and (iii) the top five predictors from the 1-year model that did not appear among the 30-day top features, thereby capturing mid-term-specific determinants. Missing features in either model were assigned a value of zero to indicate lack of importance in that model. The resulting 17-feature panel was visualized using a radar chart in RAWGraphs to depict differences in relative feature importance between short- and mid-term outcomes.

### 2.4. Statistics

Continuous variables are presented as mean ± standard deviation, and categorical variables as absolute counts (percentages). The normality of continuous variables was assessed using the Shapiro–Wilk test. Depending on the distribution, comparisons between groups were performed using Student's *t*-test or the Mann–Whitney U test for continuous variables, and the chi-squared or Fisher’s exact test for categorical variables. A *p*-value < 0.05 was considered statistically significant.

## 3. Results

A total of 581 patients who underwent OHT were included in the analysis. The 30-day and 1-year mortality rates were 9.9% and 17.6%, respectively.

In conventional groupwise analyses, several clinical and biochemical features showed significant differences between patients with versus without adverse outcomes (Table 1).

Higher age, lower serum albumin and total protein levels, as well as elevated alkaline phosphatase and γ-glutamyltransferase levels, were observed in patients who died within 30 days. Early death also occurred more frequently among patients with preoperative mechanical circulatory support or bridge-to-transplant status. Postoperative factors linked with 30-day mortality included higher norepinephrine, milrinone, and terlipressin requirements, vasoplegia, more frequent reoperations, renal dysfunction, and the need for postoperative mechanical circulatory support (Table 1).

Higher age, reduced serum albumin and total protein levels, elevated creatinine, and a lower estimated glomerular filtration rate were observed in patients with poorer one-year survival rates. Elevated C-reactive protein and alkaline phosphatase levels characterized patients with higher mortality. Postoperative factors linked with one-year death included prolonged or intensified use of vasoactive and inotropic support (norepinephrine, terlipressin, milrinone, dobutamine), vasoplegia, more frequent reoperations, renal dysfunction, dialysis requirement, abdominal dysfunction, and the need for postoperative mechanical circulatory support (Table 1). Rejection burden did not demonstrate any association with adverse postoperative outcomes.

### 3.1. ML-Based Prediction of Mortality Using Preoperative Features

**Figure 1:**
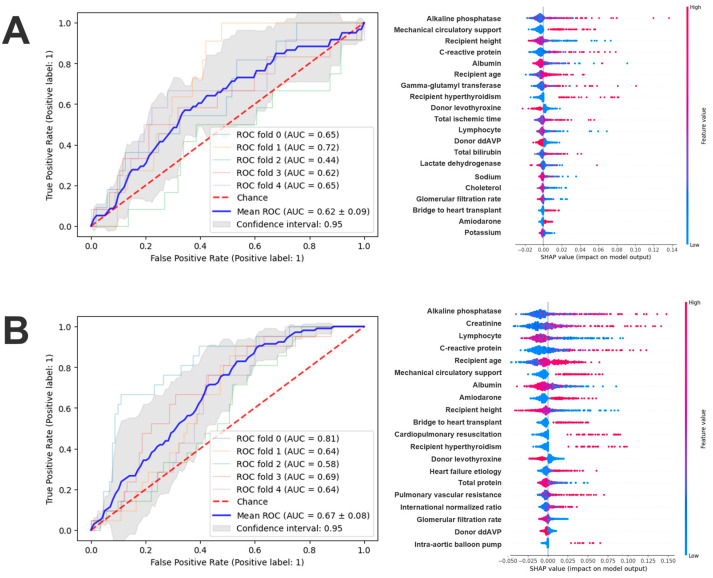
30-day (**A**) and 1-year (**B**) mortality predictions using only preoperative features. Abbreviation: ddAVP—1-desamino-8-D-arginine vasopressin.

Using preoperative features only, the random forest model achieved AUCs of 0.62 (95% CI: 0.48–0.75; Brier score: 0.09) for 30-day and 0.67 (95% CI: 0.56–0.78; Brier score: 0.15) for 1-year all-cause mortality prediction (Figure 1).

The contribution of physiological domains differed between short- and longer-term outcomes. The 30-day model was dominated by markers of acute physiological vulnerability. Alkaline phosphatase, a marker that can reflect hepatobiliary or systemic processes, was strongly associated with risk. Preoperative mechanical circulatory support and total ischemic time further underscored the importance of hemodynamic instability and procedural stress. Inflammatory and nutritional markers—C-reactive protein, albumin, and lymphocyte percentage—also ranked highly, suggesting that systemic inflammation and catabolic state predispose to early mortality. Age, serum sodium, and thyroid status provided additional prognostic value (Figure 1A and Supplementary Table S1).

**Figure 2:**
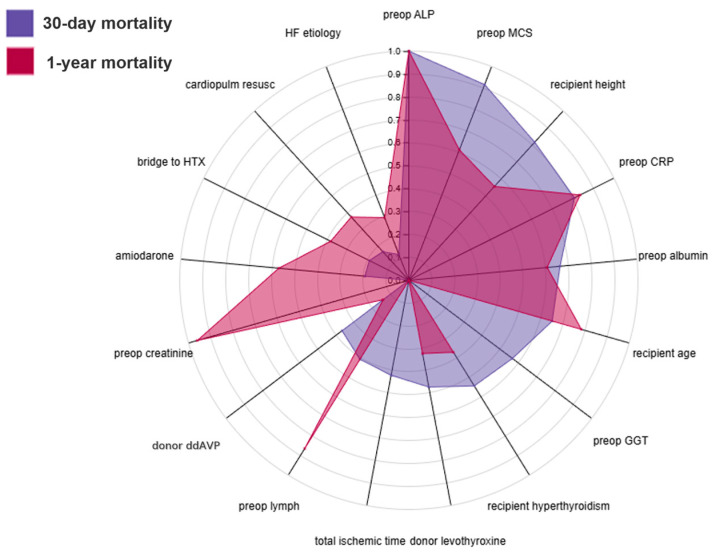
Radar plot illustrating the normalized SHAP value profiles of the 30-day and 1-year post-transplant mortality models using only preoperative predictors. Each axis represents one of the selected predictors, and the shaded polygons depict their relative contributions to short-term (blue) and mid-term (red) mortality risk. Abbreviations: ALP—alkaline phosphatase; MCS—mechanical circulatory support; CRP—C-reactive protein; ddAVP—1-desamino-8-D-arginine vasopressin; GGT—gamma-glutamyl transferase; HTX—heart transplantation; HF etiology—underlying cause of heart failure.

In the 1-year model, the emphasis shifted toward chronic organ function, metabolic reserve, and systemic homeostasis (Figure 2). Alkaline phosphatase remained the most important feature, but renal indices—notably serum creatinine and glomerular filtration rate—gained importance, highlighting the role of baseline renal function in longer-term survival. The persistent effects of inflammation (C-reactive protein) and nutritional status (albumin, total protein, lymphocyte count) indicated that metabolic frailty and immune competence continued to influence outcomes beyond the postoperative phase. Age also became a stronger determinant, consistent with the influence of frailty on late mortality. Features such as bridge-to-transplant status, donor thyroid therapy, and amiodarone use contributed additional, context-dependent prognostic information (Figure 1B and Supplementary Table S2).

### 3.2. ML-Based Prediction of Mortality Using All Preoperative and Postoperative Features

**Figure 3:**
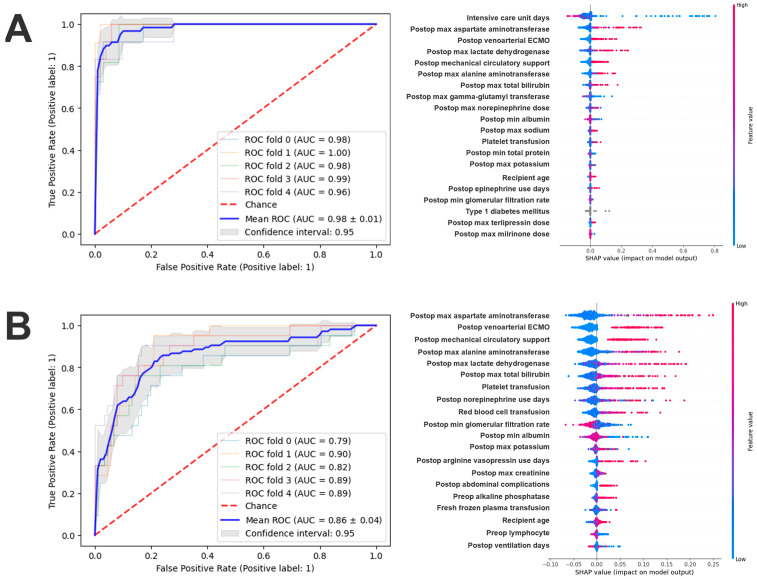
30-day (**A**) and 1-year (**B**) mortality predictions using all available (pre- and postoperative) features.

Using all available pre- and postoperative features, the random forest model achieved AUCs of 0.98 (95% CI: 0.97–0.99; Brier score: 0.02) and 0.86 (95% CI: 0.80–0.92; Brier score: 0.10) for predicting all-cause mortality at 30 days and 1 year, respectively (Figure 3).

In these models, postoperative physiological and biochemical features were the most dominant predictors of adverse outcomes. For 30-day mortality, the most important feature was the length of stay in the intensive care unit, followed by the peak values of hepatic and cellular injury markers, including maximum aspartate aminotransferase, lactate dehydrogenase, alanine aminotransferase, and total bilirubin levels. These findings indicate that early postoperative hepatic dysfunction and tissue stress strongly influenced short-term outcomes. The need for postoperative mechanical circulatory support or extracorporeal membrane oxygenation further emphasized the prognostic relevance of severe hemodynamic compromise. Electrolyte disturbances and reductions in protein concentrations, such as minimum albumin and total protein levels, provided additional information reflecting the severity of critical illness (Figure 3A and Supplementary Table S3).

**Figure 4:**
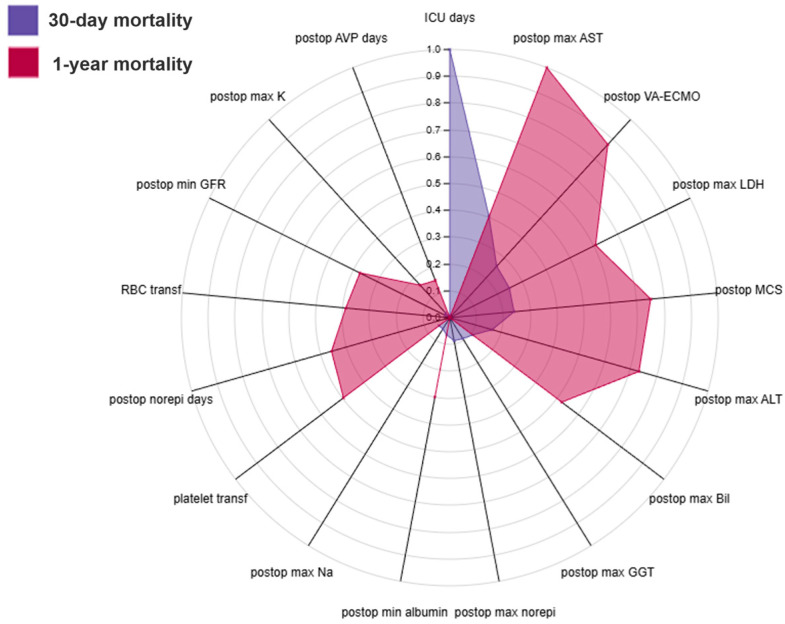
Radar plot illustrating the normalized SHAP value profiles of the 30-day and 1-year post-transplant mortality models using all available pre- and postoperative predictors. Each axis represents one of the selected predictors, and the shaded polygons depict their relative contributions to short-term (blue) and mid-term (red) mortality risk. Abbreviations: AVP days—duration of vasopressin therapy; ICU days—length of stay in the intensive care unit; AST—aspartate aminotransferase; ALT—alanine aminotransferase; LDH—lactate dehydrogenase; GGT—gamma-glutamyl transferase; Bil—total bilirubin; VA-ECMO—veno-arterial extracorporeal membrane oxygenation; MCS—mechanical circulatory support; GFR—glomerular filtration rate; Na—sodium; K—potassium; RBC transf—red blood cell transfusions; platelet transf—platelet transfusions; norepi days—duration of norepinephrine therapy.

In the 1-year model, similar postoperative features remained highly influential, but their importance shifted toward markers of persistent organ dysfunction and recovery capacity (Figure 4). Peak transaminase, lactate dehydrogenase, and bilirubin values continued to be strong predictors, suggesting that early hepatic and systemic stress responses have long-lasting prognostic implications. The need for postoperative circulatory support and the duration of vasoactive therapy were also key determinants, underscoring the long-term consequences of early postoperative instability. Measures of renal function, including minimum estimated glomerular filtration rate and peak creatinine concentration, as well as indicators of electrolyte balance and nutritional depletion, such as minimum albumin levels, further highlighted the interaction between early organ injury, metabolic resilience, and longer-term survival (Figure 3B and Supplementary Table S4).

## 4. Discussion

In this single-center, retrospective, exploratory study, we identified distinct yet interrelated patterns of risk underlying short- and mid-term mortality after OHT using preoperative and immediate postoperative data. ML models based solely on preoperative features already captured meaningful prognostic information, emphasizing the roles of hepatic, renal, inflammatory, and nutritional status in shaping postoperative outcomes. The inclusion of early postoperative features further improved predictive performance, with mortality determinants shifting toward indices reflecting the acute physiological response to surgery and early organ stress. Markers of hepatic injury, renal dysfunction, and the need for postoperative circulatory support emerged as dominant predictors, linking early postoperative instability to both short- and mid-term survival. Collectively, these findings highlight the dynamic nature of perioperative risk: while baseline physiological reserve defines vulnerability, the immediate postoperative course ultimately determines trajectory and outcome. These results underscore the potential of explainable ML approaches to enhance individualized risk assessment and guide perioperative management in heart transplantation.

ML offers new opportunities to improve prognostic modeling in transplantation by capturing nonlinear and interactive effects that traditional Cox and logistic regression-based methods cannot fully represent. In lung transplantation, Yeo et al. demonstrated that algorithms trained on a large international registry achieved excellent discrimination for 1-year mortality (AUCs up to 0.96 in internal and 0.91 in external validation), with preserved performance even when limited to pretransplant features [11]. These results emphasize that objective physiological and biochemical markers often surpass traditional diagnostic classifications in prognostic value, reinforcing the importance of integrating multidimensional data into prediction frameworks. Applied to heart transplantation, such models can refine candidate selection, improve perioperative management, and guide donor–recipient matching by identifying evolving and clinically meaningful predictors of risk.

The relevance of integrative approaches is further illustrated by the meta-analysis of Foroutan et al., in which they identified extracorporeal membrane oxygenation as the most consistent predictor of 1-year mortality after lung transplantation, whereas many conventional features, such as age or body mass index, showed limited or inconsistent associations [12]. This heterogeneity reflects the evolving nature of transplant populations and the diminishing relevance of static risk markers as clinical practices, device technologies, and immunosuppressive regimens advance. In OHT, similar dynamics exist: the increasing use of temporary mechanical circulatory support, changes in allocation policy, and improved perioperative care have reshaped the determinants of outcome. Accordingly, models that can adapt to these temporal shifts—such as ML algorithms—are essential for maintaining clinical relevance.

Several recent studies have highlighted the superiority of ML-based models over traditional risk scores in OHT. Ayers and colleagues demonstrated that ensemble algorithms combining random forests and deep neural networks outperformed logistic regression for survival prediction in the United Network for Organ Sharing (UNOS) cohort [13]. Similarly, Liou et al. benchmarked multiple survival ML methods and found that random survival forests and gradient-boosted Cox models achieved the highest accuracy, revealing that prognostic determinants shift between policy eras [14]. Yoon et al. introduced an adaptive clustering framework, Trees of Predictors, which further improved performance by accounting for population heterogeneity and temporal trends [15]. These collective findings affirm that ML methods not only offer higher predictive accuracy but also greater flexibility and adaptability to evolving clinical contexts.

Explainability remains a central challenge for clinical translation of ML models. In this study, we addressed this limitation by employing SHAP analysis to identify the most important predictors of mortality in a transparent and quantifiable manner. Unlike conventional univariable tests that assess each parameter in isolation, SHAP values quantify the contribution of each feature to the model’s predictions while considering nonlinear interactions and dependencies across multiple domains. This enables the detection of clinically meaningful patterns—such as the joint effects of renal, hepatic, and inflammatory dysfunction—that may not reach statistical significance individually but exert a combined influence on outcomes. The approach, therefore, complements and extends conventional groupwise analyses by providing a continuous, model-derived hierarchy of prognostic relevance.

Our findings align with and extend the growing body of evidence emphasizing the prognostic significance of biochemical, inflammatory, and nutritional markers in transplantation. Preoperative alkaline phosphatase consistently emerged as the strongest predictor across all models, reflecting the role of hepatobiliary function and systemic metabolic stress in determining postoperative resilience. Markers of renal function and systemic inflammation, such as creatinine, estimated glomerular filtration rate, and C-reactive protein, were among the key determinants of 1-year mortality, underscoring the long-term impact of multiorgan homeostasis on survival. Low albumin and total protein levels were predictive across the evaluation time points, confirming the link between nutritional reserve and transplant outcomes [16]. Postoperative features—particularly the need for mechanical circulatory support, duration of vasoactive therapy, and peak hepatic injury markers—captured the cumulative burden of perioperative stress and early organ dysfunction, integrating both procedural and recipient-level risks. Rejection episodes did not emerge as predictors of adverse outcomes even at 1-year follow-up in SHAP analyses, reflecting the adequacy of care and surveillance provided by regular biopsy-based monitoring.

Comparable observations have been reported by Zhou et al. and Kampaktsis et al., who identified serum albumin, ischemic time, and renal indices as key predictors of early mortality in single-center and registry-based cohorts [17,18]. Importantly, the use of SHAP in those studies, as in ours, enabled an interpretable link between complex model outputs and clinical reasoning, bridging the gap between computational performance and bedside applicability. Similarly, the interpretable neural network approach by Lisboa et al. demonstrated that transparency and predictive accuracy do not need to be mutually exclusive, reinforcing the importance of explainable artificial intelligence in ethically sensitive domains such as organ allocation [19].

Our study complements and extends these findings by demonstrating that the importance of predictors is not static but evolves over time. Early mortality is largely determined by acute injury and preoperative instability, while longer-term outcomes reflect chronic organ function and systemic resilience. This temporal shift emphasizes that prognostic modeling in transplantation should not be confined to a single time point but rather incorporate dynamic predictors that evolve with recovery and adaptation. ML models capable of recalibration and temporal validation, as emphasized by Miller et al., are particularly well suited to this task [6].

The strengths of our study include the comprehensive integration of preoperative and early postoperative data, the use of interpretable ML techniques, and the focus on temporal dynamics of mortality determinants. However, several limitations merit consideration. This was a single-center analysis, and external and prospective validations in larger, multicenter cohorts are necessary to confirm generalizability [20]. The sample size, while substantial for a single institution, limits model complexity and the exploration of underrepresented predictors. Furthermore, as with all retrospective studies, unmeasured confounders and center-specific practices may have influenced results. The dataset lacked sufficient granularity on postoperative management and donor–recipient matching patterns, which limited our ability to assess their potential additive value for transplant prognostication. Nonetheless, our findings demonstrate that even within these constraints, explainable ML can reveal clinically coherent and temporally distinct risk patterns not captured by conventional methods.

In conclusion, this study demonstrated that explainable ML, particularly random forest models interpreted with SHAP analysis, can provide nuanced insights into the determinants of short- and mid-term mortality after OHT. These approaches hold promise for refining patient selection, optimizing perioperative management, and informing allocation policy, ultimately supporting more personalized and data-driven care in heart transplantation.

## Figures and Tables

**Table 1:** Clinical, biochemical, and perioperative characteristics of the study population.

	All Patients (*n* = 581) 100%	30-Day Mortality		1-Year Mortality	
		No (*n* = 523) 90.01%	Yes (*n* = 58) 9.98%	No (*n* = 455) 78.31%	Yes (*n* = 102) 17.55%
**PREOPERATIVE PARAMETERS**					
Age (years)	50.7 ± 10.9	50.4 ± 11.1	53.9 ± 8.6 *	50.1 ± 11.1	53.5 ± 8.9 *
Sex, female *n* (%)	155 (26.67%)	142 (27.15%)	**13 (22.41%)**	118 (25.93%)	**28 (27.45%)**
Body weight (kg)	78.5 ± 14.08	78.5 ± 13.8	**79.3 ± 15.5**	78.6 ± 13.9	**78.7 ± 14.1**
Body height (cm)	173.9 ± 9.01	174.1 ± 9.0	**171.7 ± 8.9**	174.3 ± 9.1	172.3 ± 9.1 *
Body Mass Index	25.9 ± 4.1	25.8 ± 4.1	**26.7 ± 4.3**	25.8 ± 4.1	**26.4 ± 4.0**
NYHA class	3.5 ± 0.6	3.5 ± 0.6	**3.5 ± 0.6**	3.5 ± 0.6	**3.5 ± 0.5**
**Diagnosis leading to HTX**					
ischemic cardiomyopathy *n* (%)	375 (64.54%)	339 (64.81%)	**36 (62.06%)**	302 (66.37%)	**59 (57.84%)**
dilated cardiomyopathy *n* (%)	137 (23.58%)	125 (23.9%)	**12 (20.68%)**	107 (23.51%)	**26 (25.49%)**
restrictive cardiomyopathy *n* (%)	14 (2.40%)	12 (2.29%)	**2 (3.44%)**	7 (1.53%)	7 (6.86%) *
other causes *n* (%)	55 (9.4%)	47 (8.98%)	**8 (13.79%)**	39 (8.57%)	**10 (9.8%)**
**Recipient’s medical history**					
**Preoperative comorbidities**					
COPD *n* (%)	67 (11.53%)	63 (12.04%)	**4 (6.89%)**	55 (12.08%)	**11 (10.78%)**
Hypertension *n* (%)	266 (45.78%)	235 (44.93%)	**30 (51.72%)**	207 (45.49%)	**51 (50%)**
Diabetes mellitus *n* (%)	128 (22.03%)	116 (22.17%)	**13 (22.41%)**	99 (21.75%)	**25 (24.5%)**
Smoking *n* (%)	147 (25.3%)	138 (26.38%)	**10 (17.24%)**	124 (27.25%)	**20 (19.6%)**
Asthma *n* (%)	20 (3.4%)	19 (3.63%)	**1 (1.72%)**	17 (3.73%)	**3 (2.9%)**
Hypothyroidism *n* (%)	80 (13.76%)	76 (14.53%)	**4 (6.89%)**	67 (14.72%)	**9 (8.82%)**
Hyperthyroidism *n* (%)	31 (5.33%)	22 (4.2%)	9 (15.51%) *	18 (3.95%)	**12 (11.76%)**
Prior cerebrovascular accident *n* (%)	73 (12.56%)	63 (12.04%)	**10 (17.24%)**	53 (11.64%)	**16 (15.68%)**
Prior malignancy *n* (%)	30 (5.1%)	27 (5.16%)	**3 (5.17%)**	21 (4.6%)	**7 (6.86%)**
Preoperative autoimmune diseases *n* (%)	41 (7.05%)	36 (6.88%)	**5 (8.6%)**	33 (7.2%)	**8 (7.84%)**
Diabetes mellitus Type I *n* (%)	4 (0.68%)	2 (0.38%)	2 (3.44%) *	2 (0.43%)	**2 (1.9%)**
Hashimoto’s thyroiditis *n* (%)	5 (0.86%)	5 (0.95%)	**0 (0%)**	5 (1.09%)	**0 (0%)**
Rheumatoid arthritis *n* (%)	3 (0.51%)	3 (0.57%)	**0 (0%)**	2 (0.43%)	**1 (0.98%)**
Psoriasis *n* (%)	9 (1.54%)	8 (1.52%)	**1 (1.72%)**	7 (1.53%)	**2 (1.9%)**
Inflammatory bowel disease (IBD) *n* (%)	8 (1.37%)	8 (1.52%)	**0 (0%)**	8 (1.75%)	**0 (0%)**
Celiac disease *n* (%)	4 (0.68%)	4 (0.76%)	**0 (0%)**	4 (0.87%)	**0 (0%)**
Dermato-polymyositis *n* (%)	2 (0.34%)	1 (0.19%)	**1 (1.72%)**	1 (0.21%)	**1 (0.98%)**
Scleroderma *n* (%)	2 (0.34%)	2 (0.38%)	**0 (0%)**	1 (0.21%)	**1 (0.98%)**
**Immediate preoperative factors**					
Total Assistance with ADLs *n* (%)	63 (10.84%)	56 (10.7%)	**7 (12.06%)**	48 (10.54%)	**12 (11.76%)**
Recent Infection requiring Intravenous Antibiotics *n* (%)	99 (17.03%)	89 (17.01%)	**10 (17.24%)**	82 (18.02%)	**15 (14.7%)**
Hospitalized *n* (%)	199 (34.25%)	180 (34.41%)	**19 (32.75%)**	159 (34.94%)	**34 (33.33%)**
Ventilator-dependent *n* (%)	32 (5.5%)	29 (5.5%)	**3 (5.17%)**	25 (5.49%)	**7 (6.86%)**
Need for preoperative mechanical circulatory support *n* (%)	89 (15.31%)	70 (13.38%)	19 (32.75%) *	56 (12.30%)	30 (29.41%) *
Bridge to heart transplantation *n* (%)	78 (13.42%)	64 (12.23%)	14 (24.13%) *	51 (11.20%)	24 (23.52%) *
**Right heart catheterization**					
Pulmonary vascular resistance PVR (WU)	2.5 ± 1.2	2.5 ± 1.2	**2.7 ± 1.2**	2.5 ± 1.2	**2.7 ± 1.3**
Pulmonary artery systolic pressure PAPs (Hgmm)	41.8 ± 13.7	41.5 ± 13.5	**44.2 ± 14.7**	41.4 ± 13.4	**44.2 ± 15.0**
Pulmonary artery diastolic pressure PAPd (Hgmm)	20.1 ± 8.1	20.2 ± 8.2	**19.4 ± 7.0**	20.1 ± 8.0	**20.8 ± 8.7**
Pulmonary artery mean pressure PAPm (Hgmm)	28.4 ± 9.7	28.4 ± 9.8	**27.9 ± 8.9**	28.2 ± 9.5	**29.3 ± 10.5**
Pulmonary Artery Wedge Pressure PAWp (Hgmm)	18.8 ± 7.3	18.8 ± 7.4	**18.7 ± 6.5**	18.8 ± 7.4	**19.3 ± 7.0**
Cardiac output CO (L/min)	4 ± 1.38	4.0 ± 1.4	**3.8 ± 1.0**	4.0 ± 1.4	**4.0 ± 1.0**
**Preoperative laboratory parameters**					
INR	1.8 ± 1.7	1.8 ± 1.7	**1.8 ± 0.8**	1.8 ± 1.9	**1.8 ± 0.8**
GFR	65.4 ± 33.7	66.1 ± 34.2	**59.6 ± 28.3**	66.9 ± 33.4	57.4 ± 31.2 *
Creatinine (mg/dL)	1.3 ± 0.8	1.3 ± 0.8	**1.4 ± 0.8**	1.3 ± 0.8	1.6 ± 1.1 *
AST (GOT) (U/L)	34.8 ± 61.8	35.2 ± 64.9	**31.4 ± 23.8**	35.4 ± 69.1	**30.7 ± 22.3**
ALT (GPT) (U/L)	34.1 ± 113.9	34.8 ± 119.8	**28.1 ± 27.3**	35.4 ± 128.4	**26.4 ± 22.9**
GGT (U/L)	110.6 ± 113.5	107.1 ± 111.2	140.6 ± 129.2 *	107.1 ± 111.8	**128.8 ± 126.4**
LDH (U/L)	464.1 ± 478.9	451.5 ± 353.9	**579.7 ± 1096**	453.7 ± 358.2	**539.4 ± 819.1**
Total bilirubin (umol/L)	16.8 ± 13.5	16.4 ± 13.2	**19.9 ± 15.5**	16.5 ± 13.6	**18.3 ± 14.4**
protein (g/L)	67.9 ± 9.9	68.1 ± 9.9	**62.2 ± 10.1**	68.3 ± 9.5	66.6 ± 9.4 *
albumin (g/L)	41.8 ± 7.1	42.1 ± 7.0	39.7 ± 7.9 *	42.3 ± 6.9	39.9 ± 7.3 *
ALP (U/L)	104.1 ± 58.3	101.6 ± 54.4	125.3 ± 81.9 *	100 ± 52.3	119.7 ± 75.6 *
Sodium (mmol/L)	135.5 ± 4.58	135.5 ± 4.4	**135.2 ± 5.4**	135.5 ± 4.4	**135.1 ± 5.3**
Potassium (mmol/L)	4.3 ± 0.55	4.3 ± 0.55	**4.2 ± 0.5**	4.3 ± 0.5	**4.3 ± 0.5**
CRP (mg/L)	15.9 ± 32.7	14.2 ± 28.6	30.1 ± 54.5 *	13.1 ± 26.7	26.9 ± 48.5 *
WBC (G/L)	8.6 ± 3.2	8.5 ± 3.1	**8.7 ± 3.7**	8.5 ± 2.9	**9.1 ± 3.9**
lymphocytes (%)	17.9 ± 8.4	18.2 ± 8.4	**15.6 ± 7.9**	18.6 ± 8.5	15.4 ± 7.4 *
**Donor parameters**					
Age (years)	41.1 ± 11.4	41.1 ± 11.5	**41.2 ± 10.6**	41.0 ± 11.5	**40.8 ± 10.9**
Sex, female n (%)	155 (26.81%)	139 (26.57%)	**16 (27.58%)**	117 (25.71%)	**31 (30.39%)**
Body weight (kg)	83.3 ± 15.1	83.1 ± 15.0	**84.5 ± 16.1**	83.5 ± 14.8	**82.8 ± 15.9**
Body height (cm)	176.1 ± 8.6	176.2 ± 8.6	**175.3 ± 8.7**	176.4 ± 8.6	**175.1 ± 8.6**
Body Mass Index	26.5 ± 4.8	26.4 ± 4.9	**27.2 ± 4.3**	26.4 ± 5.0	**26.8 ± 4.3**
Received Solu-Medrol *n* (%)	151 (25.98%)	139 (26.57%)	**12 (20.68%)**	123 (27.03%)	**18 (17.64%)**
Received ddAVP *n* (%)	296 (50.94%)	274 (52.39%)	22 (37.93%) *	237 (52.08%)	42 (41.17%) *
Received Levothyroxin *n* (%)	198 (34.07%)	187 (35.75%)	11 (18.96%) *	169 (37.14%)	**25 (24.5%)**
Total ischemic time during heart transplantation (min)	192 ± 53.4	191.1 ± 50.7	**205.3 ± 73.0**	191.1 ± 49.8	**196.5 ± 66.5**
**POSTOPERATIVE PARAMETERS**					
**Postoperative maximum values of laboratory parameters**					
AST (GOT) (U/L)	282.8 ± 913.3	135.0 ± 184.0	1760 ± 2559 *	124.6 ± 159.3	1059.2 ± 2037.6 *
ALT (GPT) (U/L)	163.6 ± 542.7	84.2 ± 193.2	955.8 ± 1486.2 *	74.3 ± 160.4	602.2 ± 1184.7 *
GGT (U/L)	207.7 ± 248.9	218.0 ± 255.9	106.6 ± 127.3 *	217.4 ± 260.0	**160.4 ± 204.3**
LDH (U/L)	1211.1 ± 1640.4	932.3 ± 529.1	3971.2 ± 4300.7 *	917.7 ± 454.1	2747.5 ± 3497.0 *
Total bilirubin (umol/L)	35.1 ± 29.2	32.4 ± 24.8	62.6 ± 49.7 *	31.4 ± 23.4	55.7 ± 44.7 *
ALP (U/L)	133.4 ± 120.3	135.6 ± 122.9	**107.2 ± 78.4**	131.7 ± 112.4	**152.9 ± 169.7**
Sodium (mmol/L)	144.8 ± 6.1	144.4 ± 6.1	148.6 ± 5.3 *	144.4 ± 6.2	152.9 ± 169.7 *
Potassium (mmol/L)	5.4 ± 0.6	5.4 ± 0.5	5.8 ± 0.9 *	5.4 ± 0.5	5.7 ± 0.8 *
Creatinine (mg/dL)	1.72 ± 1	1.6 ± 1.04	2.1 ± 0.8 *	1.6 ± 1.0	2.1 ± 1.1 *
**Postoperative minimum values of laboratory parameters**					
GFR	53.2 ± 28.8	54.5 ± 29.1	39.2 ± 22.4 *	55.5 ± 28.7	38.0 ± 21.3 *
Protein (g/L)	36.4 ± 9.2	37.1 ± 9.1	29.6 ± 8.2 *	37.1 ± 8.1	32.7 ± 86.6 *
Albumin (g/L)	29.23 ± 5.4	26.6 ± 4.8	24.5 ± 8.4 *	29.7 ± 4.8	26.7 ± 7.4 *
**Circulatory support medications**					
norepinephrine use *n* (%)	533 (91.73%)	487 (93.11%)	46 (79.31%) *	423 (92.96%)	88 (86.27%) *
norepinephrine max dose (ug/kg/min)	0.47 ± 10.5	0.44 ± 0.4	0.76 ± 0.7 *	0.42 ± 0.4	0.7 ± 0.6 *
norepinephrine use days	6.51 ± 9.7	6.5 ± 9.9	**6.0 ± 7.5**	5.4 ± 7.5	11.8 ± 15.9 *
epinephrine use *n* (%)	240 (41.3%)	213 (40.72%)	**27 (46.55%)**	183 (40.21%)	**53 (51.96%)**
epinephrine max dose (ug/kg/min)	0.05 ± 0.5	0.05 ± 0.5	**0.05 ± 0.1**	0.05 ± 0.6	**0.04 ± 0.1**
epinephrine use days	1.74 ± 3.5	1.6 ± 3.1	2.8 ± 5.6 *	1.5 ± 3.0	2.9 ± 5.2 *
milrinone use *n* (%)	533 (91.73%)	488 (93.3%)	45 (77.58%) *	424 (93.18%)	**87 (85.29%)**
milrinone max dose (ug/kg/min)	0.37 ± 0.2	0.38 ± 0.2	**0.32 ± 0.3**	0.37 ± 0.2	**0.35 ± 0.2**
milrinone use days	7.9 ± 8	8.2 ± 8.1	5.3 ± 6.4 *	7.5 ± 6.1	9.6 ± 13.7 *
vasopressine use *n* (%)	156 (26.85%)	139 (26.57%)	**17 (29.31%)**	113 (24.83%)	**29 (28.43%)**
vasopressin use days	1.38 ± 3.83	1.37 ± 3.8	**1.7 ± 4.6**	1.0 ± 2.1	2.9 ± 7.6 *
terlipressine use *n* (%)	173 (29.77%)	146 (27.91%)	27 (46.55%) *	130 (28.57%)	43 (42.15%) *
terlipressine max dose (ug/kg/min)	0.38 ± 0.69	0.34 ± 0.6	0.7 ± 0.9 *	0.34 ± 0.6	0.62 ± 0.9 *
terlipressine use days	1.5 ± 4.5	1.3 ± 4.3	3.0 ± 6.0 *	1.2 ± 4.0	3.1 ± 6.4 *
dobutamine use *n* (%)	423 (72.8%)	389 (74.37%)	**35 (60.34%)**	334 (73.40%)	**68 (66.66%)**
dobutamine max dose (ug/kg/min)	4.07 ± 3.1	4.1 ± 3.0	**3.5 ± 3.6**	3.5 ± 2.7	**4.3 ± 4.0**
dobutamine use days	4.38 ± 5.7	4.4 ± 5.8	**3.7 ± 5.2**	3.9 ± 2.9	5.8 ± 8.6 *
**Total number of blood transfusions during hospitalization**					
Red blood cell concentrate (units)	14.7 ± 18.9	14.0 ± 19.1	20.8 ± 16.1 *	11.9 ± 15.4	27.3 ± 27.4 *
Fresh Frozen plasma (units)	6.4 ± 11.4	6.0 ± 11.4	10.4 ± 10.2 *	5.1 ± 7.7	12.3 ± 20.4 *
Platelet concentrate (units)	10.6 ± 18.1	9.4 ± 16.1	22 ± 28.7 *	8.1 ± 14.5	19.8 ± 27.0 *
**Postoperative clinical data and complications**					
ICU days *n* (%)	19.7 ± 23.4	21.2 ± 24.1	6.4 ± 7.6 *	18.6 ± 19.7	25.7 ± 36.9 *
Ventilation days *n* (%)	10.3 ± 21	10.8 ± 22.8	**6.1 ± 7.3**	8.4 ± 18.1	20.0 ± 30.9 *
Underwent tracheostomy *n* (%)	82 (14.11%)	76 (14.53%)	**6 (10.34%)**	50 (10.98%)	**31 (30.39%)**
Vasoplegia occured *n* (%)	114 (19.62%)	93 (17.78%)	21 (36.2%) *	80 (17.58%)	32 (31.37%) *
CPR occured *n* (%)	30 (5.16%)	24 (4.5%)	6 (10.34%) *	18 (3.95%)	12 (11.76%) *
Heart retransplantation *n* (%)	7 (1.20%) n = 397	6 (1.14%)	**1 (1.72%)**	4 (0.87%)	**2 (1.9%)**
Reoperation *n* (%)	99 (17.03%)	79 (15.01%)	20 (34.48%) *	61 (13.40%)	35 (34.31%) *
Renal dysfunction *n* (%)	178 (30.63%)	150 (28.68%)	28 (48.27%) *	120 (26.37%)	52 (50.98%) *
Need for dialysis *n* (%)	140 (24.09%)	117 (22.37%)	23 (39.65%) *	88 (19.34%)	45 (44.11%) *
Infection *n* (%)	216 (37.17%)	199 (38.04%)	**17 (29.31%)**	164 (36.04%)	**47 (46.07%)**
Neurological dysfunction *n* (%)	29 (5%)	25 (4.78%)	**4 (6.89%)**	19 (4.17%)	**9 (8.82%)**
Rejection *n* (%)	116 (19.96%)	112 (21.41%)	**4 (6.89%)**	97 (21.31%)	**18 (17.64%)**
Abdominal dysfunction *n* (%)	62 (10.67%)	49 (9.36%)	13 (22.41%) *	33 (7.25%)	28 (27.45%) *
Thyroid dysfunction *n* (%)	74 (0.12%)	67 (12.8%)	**9 (15.51%)**	57 (12.52%)	**15 (14.7%)**
Need for postoperative mechanical circulatory support *n* (%)	111 (19.1%)	70 (13.38%)	41 (70.68%) *	48 (10.54%)	59 (57.58%) *

Variables are presented as mean ± standard deviation or number (percentage), as appropriate. Statistically significant differences between survivors and non-survivors (*p* < 0.05) are indicated by an asterisk (*). Abbreviations: NYHA—New York Heart Association functional class; PVR—pulmonary vascular resistance; WU—Wood units; PAPs/PAPd/PAPm—pulmonary artery systolic/diastolic/mean pressure; PAWP—pulmonary artery wedge pressure; CO—cardiac output; INR—international normalized ratio; GFR/eGFR—(estimated) glomerular filtration rate; AST (GOT)—aspartate aminotransferase; ALT (GPT)—alanine aminotransferase; ALP—alkaline phosphatase; GGT—γ-glutamyltransferase; LDH—lactate dehydrogenase; CRP—C-reactive protein; WBC—white blood cell count; ICU—intensive care unit; CPR—cardiopulmonary resuscitation; ADL(s)—activities of daily living; ddAVP—1-desamino-8-D-arginine vasopressin; IBD—inflammatory bowel disease.

## Data Availability

The original contributions presented in this study are included in the article/Supplementary Materials. Further inquiries can be directed to the corresponding author.

## Supplementary Materials

The following supporting information can be downloaded at: https://www.mdpi.com/article/10.3390/jcdd12120486/s1. Table S1. Feature Importances (Descending order; Non-zero Only). 30-day mortality, preoperative features only; Table S2. Feature Importances (Descending order; Non-zero Only). 1-year mortality, preoperative features only; Table S3. Feature Importances (Descending order; Non-zero Only). 30-day mortality, all pre- and postoperative variables; Table S4. Feature Importances (Descending order; Non-zero Only). 1-year mortality, all pre- and postoperative variables
