# Supplementary material for: Shifting Determinants of Mortality Risk After Orthotopic Heart Transplantation Identified by Machine Learning
Source: J Cardiovasc Dev Dis. 2025 Dec 10;12(12):486. doi: 10.3390/jcdd12120486 (PMC12734402; doi:10.3390/jcdd12120486)
Supplement: Supplementary file 1 [file jcdd-12-00486-s001.zip › jcdd-3989055-supplementary.pdf]

Shifting Determinants of Mortality Risk After Orthotopic Heart Transplantation Identified by Machine Learning

Supplementary Tables

Feature Importances (Descending order; Non-zero Only)

Supplementary Table S1. 30-day mortality, preoperative features only

| **feature** | **Mean absolute SHAP value** |
|---|---|
| Alkaline phosphatase | 0.007500 |
| Mechanical circulatory support | 0.006895 |
| Recipient height | 0.006124 |
| C-reactive protein | 0.006055 |
| Albumin | 0.004990 |
| Recipient age | 0.004887 |
| Gamma-glutamyl transferase | 0.004251 |
| Recipient hyperthyroidism | 0.004073 |
| Donor levothyroxine | 0.003576 |
| Total ischemic time | 0.003169 |
| Lymphocyte | 0.003041 |
| Donor ddAVP | 0.002737 |
| Total bilirubin | 0.002681 |
| Lactate dehydrogenase | 0.002008 |
| Sodium | 0.001798 |

Supplementary Table S2. 1-year mortality, preoperative features only

| **feature** | **Mean absolute SHAP value** |
|---|---|
| Alkaline phosphatase | 0.017075 |
| Creatinine | 0.016421 |
| Lymphocyte | 0.014753 |
| C-reactive protein | 0.014296 |
| Recipient age | 0.013408 |
| Mechanical circulatory support | 0.010431 |
| Albumin | 0.010408 |
| Amiodarone | 0.009733 |
| Recipient height | 0.009508 |
| Bridge to heart transplant | 0.006545 |
| Cardiopulmonary resuscitation | 0.006397 |
| Recipient hyperthyroidism | 0.006339 |
| Donor levothyroxine | 0.005608 |
| Heart failure etiology | 0.005040 |
| Total protein | 0.004926 |

Supplementary Table S3. 30-day mortality, all pre- and postoperative variables

| feature | Mean absolute SHAP value |
|---|---|
| Intensive care unit days | 0.060137 |
| Postop max aspartate aminotransferase | 0.024298 |
| Postop venoarterial ECMO | 0.015611 |
| Postop max lactate dehydrogenase | 0.014921 |
| Postop mechanical circulatory support | 0.014447 |
| Postop max alanine aminotransferase | 0.009770 |
| Postop max total bilirubin | 0.006509 |
| Postop max gamma-glutamyl transferase | 0.005576 |
| Postop max sodium | 0.005285 |
| Postop min albumin | 0.003844 |
| Postop max sodium | 0.003064 |
| Platelet transfusion | 0.002967 |
| Postop min total protein | 0.002362 |
| Postop max potassium | 0.001393 |
| Recipient age | 0.001289 |

Supplementary Table S4. 1-year mortality, all pre- and postoperative variables

| **feature** | **Mean absolute SHAP value** |
|---|---|
| Postop max aspartate aminotransferase | 0.031835 |
| Postop venoarterial ECMO | 0.027910 |
| Postop mechanical circulatory support | 0.024060 |
| Postop max alanine aminotransferase | 0.023387 |
| Postop max lactate dehydrogenase | 0.019439 |
| Postop max total bilirubin | 0.016826 |
| Platelet transfusion | 0.015930 |
| Postop epinephrine use days | 0.014784 |
| Red blood cell transfusion | 0.012612 |
| Postop min glomerular filtration rate | 0.012056 |
| Postop min albumin | 0.009847 |
| Postop max potassium | 0.005400 |
| Postop arginine vasopressin use days | 0.004985 |
| Postop max creatinine | 0.003972 |
| Postop abdominal complications | 0.003872 |
